# Brain–Computer Interfacing Using Functional Near-Infrared Spectroscopy (fNIRS)

**DOI:** 10.3390/bios11100389

**Published:** 2021-10-13

**Authors:** Kogulan Paulmurugan, Vimalan Vijayaragavan, Sayantan Ghosh, Parasuraman Padmanabhan, Balázs Gulyás

**Affiliations:** 1 Cognitive Neuroimaging Centre, 59 Nanyang Drive, Nanyang Technological University, Singapore 636921, Singapore; paulmurugankogulan@gmail.com (K.P.); balazs.gulyas@ntu.edu.sg (B.G.); 2 Department of Integrative Biology, Vellore Institute of Technology, Vellore 632014, India; sayantan7@gmail.com; 3 Imaging Probe Development Platform, 59 Nanyang Drive, Nanyang Technological University, Singapore 636921, Singapore; 4 Department of Clinical Neuroscience, Karolinska Institute, 17176 Stockholm, Sweden

**Keywords:** functional near-infrared spectroscopy (fNIRS), non-invasive monitoring, brain function, neuron function, blood oxygen concentration, cognitive function, brain–computer interfacing, current advancement

## Abstract

Functional Near-Infrared Spectroscopy (fNIRS) is a wearable optical spectroscopy system originally developed for continuous and non-invasive monitoring of brain function by measuring blood oxygen concentration. Recent advancements in brain–computer interfacing allow us to control the neuron function of the brain by combining it with fNIRS to regulate cognitive function. In this review manuscript, we provide information regarding current advancement in fNIRS and how it provides advantages in developing brain–computer interfacing to enable neuron function. We also briefly discuss about how we can use this technology for further applications.

## 1. Introduction

A brain–computer interface (BCI) is a system that acquires signals from the brain, translates the signals, and outputs to devices in order to enact a desired action [1]. A BCI system is composed of both hardware and software components, and in general, is executed in five steps, viz., signal acquisition, pre-processing, feature extraction, feature translation, and device output. BCI systems are classified into several types based on the functional imaging systems they are interfaced with, such as Electroencephalography (EEG)-BCI, functional Magnetic Resonance Imaging (fMRI)-BCI, and functional Near-Infrared Spectroscopy (fNIRS)-BCI. In this review, we discuss in detail the BCI based on fNIRS and how it functions, the advantages and disadvantages of its utilities, and its application and implementation in useful technology, and the future of fNIRS-BCI.

**Figure 1:**
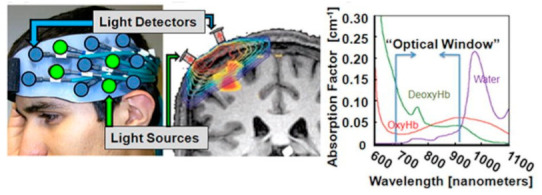
Example of how an emitter–detector pair works along with the optical window looked at to refine the data. (Reprinted with permission from ref. [6] (Copyright 2012 Elsevier)). Available online: https://www.sciencedirect.com/science/article/abs/pii/S0966636211004115?via%3Dihub (accessed on 9 September 2021).

Functional Near-Infrared Spectroscopy (fNIRS) is an optical imaging technique in which the emitted light in the brain undergoes attenuation due to absorption and scattering. It utilizes the general transparency property of the bones and skin to gain access to the tissues that are being monitored. While the absorbed light contributes to the inside of the medium where it is absorbed, the detector measures the non-absorbed component of the scattered light (Figure 1). As a result of the hemodynamic response due to the given stimuli, there is an increase and decrease in oxyhemoglobin (OxyHb) and deoxyhemoglobin (deoxyHb), respectively. When the light is emitted, the regional changes in the hemodynamic response result in regional changes in the light absorption and the absorption spectra of the chromophores, thereby allowing the quantification of the oxyHb and deoxyHb in a non-invasive fashion by using the Beer–Lambert law [2,3]. The ratio of the concentrations of oxyHb as a result of the arterial flow and deoxyHb in venous blood flow against total hemoglobin (tHb) is determined by calculating this ratio [4]. fNIRS is a non-invasive and portable device, allowing it a wider degree of freedom for use. By monitoring a selective region of the brain that exhibits activity when a passive or active stimulation occurs (i.e., tapping a finger or flashing lights or performing cognitive tasks), we can attribute the change in hemoglobin (Hb) concentration to the neuronal activity. Since cranial bones block lights within the visible range, fNIRS utilizes near-infrared light (650–1000 nm) to monitor and detect the changes in oxyHb and deoxyHb in the brain [5].

**Figure 2:**
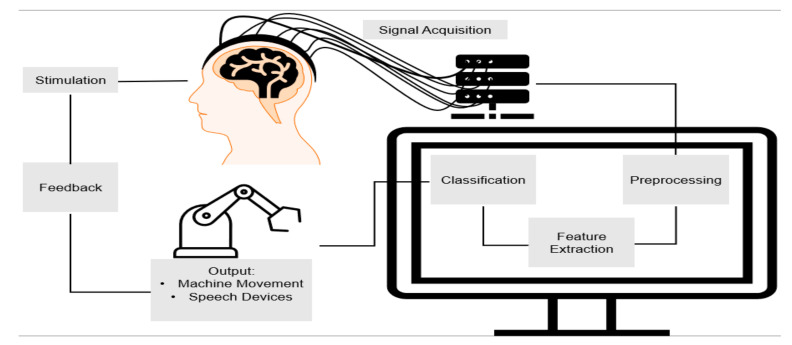
The process of brain–computer interfaces. The cycle begins with a stimulation that triggers brain activity. The activity is acquired in a variety of ways and sent to a computer for pre-processing. After processing the data, certain features are extracted, classified, and an output is determined and sent. The cycle begins anew after the output creates feedback of a new stimulation.

The concept of fNIRS relies on the fact that certain wavelengths of light can penetrate the skin and skull (Figure 2) [3]. In the experimental setup, the patient wears a fNIRS cap that consists of sources and detectors. The sources emit near-infrared light at a particular wavelength while the detectors read the reflected and refracted light at shifted wavelengths. For a deeper imaging, the distance between the emitter and detector must be increased, but as the distance increases, the spatial resolution diminishes [7]. The number of emitters and detectors and the montage are determined based on the prior knowledge of the activation pattern for the given stimuli.

**Figure 3:**
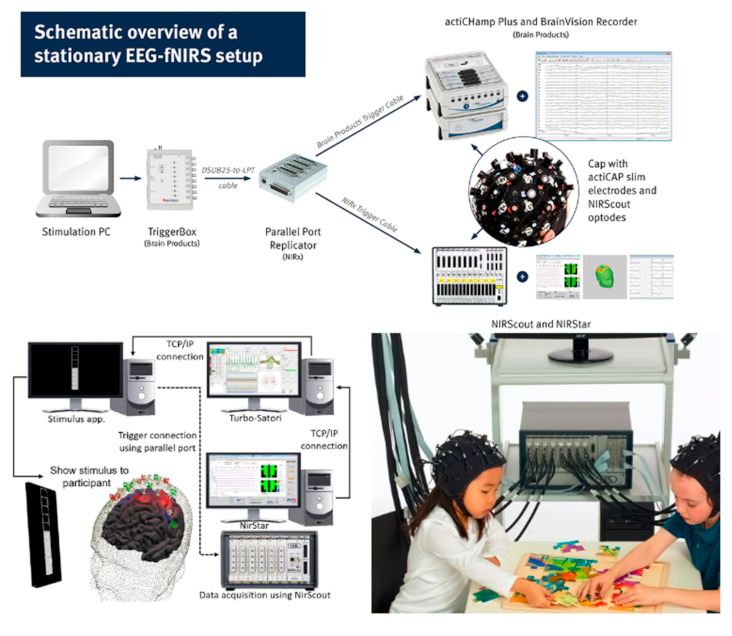
Schematic overview of a stationary EEG–fNIRS setup. (Reprinted with permission from Brain Products Press Release. Copyright 2020. Available online: https://pressrelease.brainproducts.com/category/2020/ accessed on 10 July 2021).

The development of fNIRS started in 1992, and the hardware has only been improved since then. Here, we present a brief history of the important developments of fNIRS hardware over the years (Table 1). The use of near-infrared spectroscopy (NIRS) to detect hemoglobin was first reported by Frans Jöbsis in 1977 (https://www.artinis.com/theory-of-nirs (accessed on 10 July 2021)) [1]. The technology has moved on far from its humble beginnings, where other modalities such as EEG and fMRI are being used in conjunction with fNIRS setups and the systems are moving towards commercialization (Figure 3).

**Figure 4:**
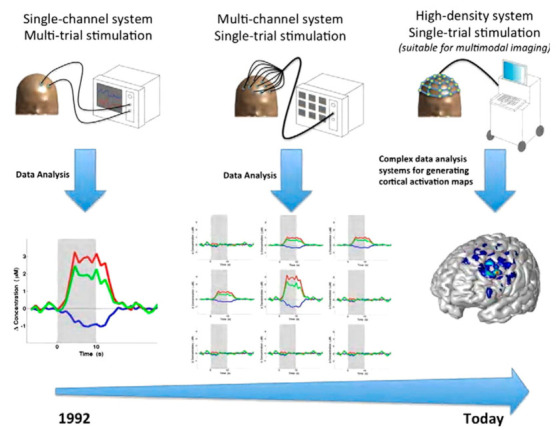
The development of fNIRS imaging over time. (Reprinted with permission from ref. [1], Copyright 2012 Elsevier).

The original equipment was designed by Jöbsis as a single-channel continuous wave (CW) system and had low spatial resolution and was utilized for qualitative purposes, and later versions were developed with enhanced resolution [4]. The use of NIRS machines to obtain changes in HbO and HbR levels was first developed in 1984 by David Delphy. By 1989, the first commercial system was built by Hamamatsu Photonics. Up until 1993, most of the experiments utilized a single-node system. Then, Hoshi and Tamura demonstrated the usefulness of multiple channels by using five single-channel fNIRS machines. This spurred the creation of the 10-channel CW system by Hitachi in 1994. By 2004, the 64-channel and 52-channel were the most common types of fNIRS used in various applications (Figure 4). The first compact wireless systems were four-channel systems that were sold by fNIRS Devices in 2011. In late 2011, a wearable, wireless battery-operated single-channel system was introduced by Artinis. The commercial fNIRS system can be used to measure the hemodynamic response in a resting state or under a challenge condition [8,9].

**Figure 5:**
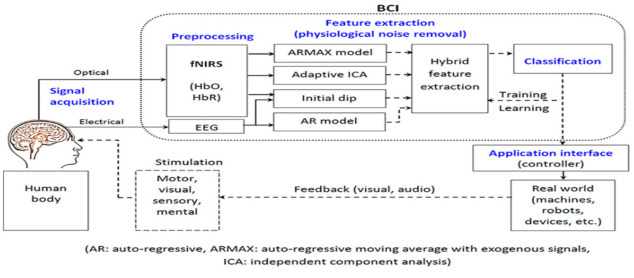
An example of a combined fNIRS and EEG brain–computer interface system. (Reprinted from [10]).

The focus of this manuscript is to discuss the role of fNIRS-BCI design and application in the regulation of neuron function [3,9]. By using the hemodynamic response and acquisition of data by the fNIRS system, it is possible to translate the data to execute a desired physical activity. Like all BCI systems, fNIRS-BCI follows the same pathway of signal acquisition, pre-processing, feature extraction, and output (Figure 5). 

## 2. Steps in Implementing fNIRS-BCI

**Figure 6:**
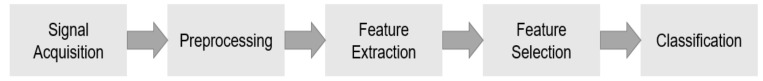
The track an fNIRS-BCI takes in order to receive useable data for an output and the order we will be following to understand the fNIRS-BCI process.

The general steps in implementing BCI comprise: (1) acquiring the brain signals, (2) pre-processing the acquired data, (3) feature extraction relevant to the nature of the acquired brain signals, (4) feature selection, and (5) classification. Relationships between each of these steps are outlined in Figure 6.

**Figure 7:**
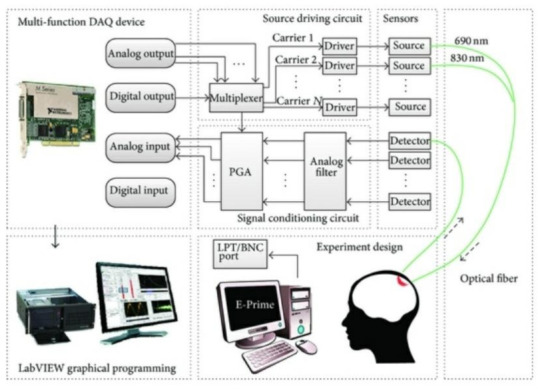
An example of a combined continuous wave and fNIRS brain–computer interface system. (Reprinted from [11]).

Correlations between these five steps have been discussed earlier in the text and shown (Figure 2). The mandatory steps are data acquisition, pre-processing, and feature selection and they need to be a part of every BCI setup, whereas the optional approaches are machine learning and AI usages along with continuous data analytics and decision-making approaches to make the setup more reliable, automated, and accurate. These steps together form the extract, transform, and load (ETL) layer of a BCI setup (Figure 7).

### 2.1. Data Acquisition

**Figure 8:**
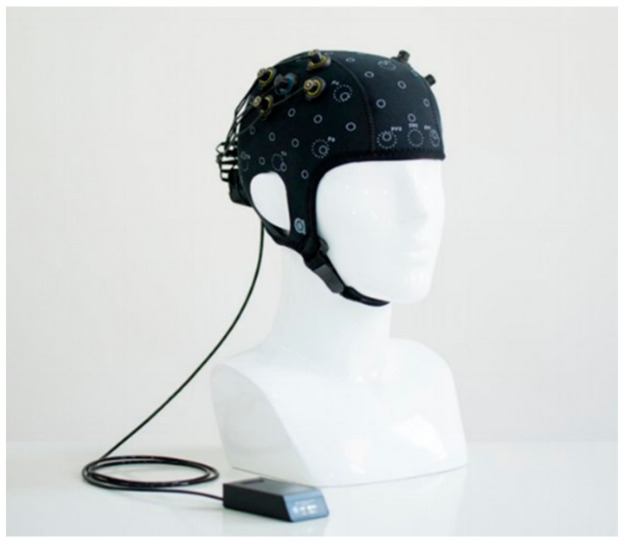
An fNIRS imaging cap designed and built by Artinis for use of monitoring the hemodynamic response. (Reprinted from Artinis OctaMon, with permission from Artinis Medical Systems. Copyright 2021. Available online: https://www.artinis.com/octamon accessed on 12 September 2021).

**Figure 9:**
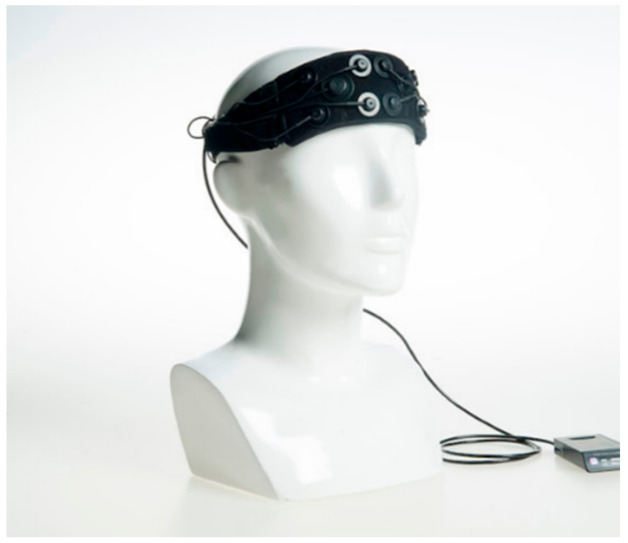
An eight-channel fNIRS device built by Artinis for imaging of the prefrontal cortex. (Reprinted from Artinis OctaMon, with permission from Artinis Medical Systems. Copyright 2021. Available online: https://www.artinis.com/octamon accessed on 12 September 2021).

The acquisition of the hemodynamic response from the brain is the first step in the process of obtaining the necessary data. The underlying principle of fNIRS relies on neurovascular coupling and the Blood Oxygen Dependent (BOLD) response as a result of a passive or active stimulus. In a typical fNIRS experimental setup, a predetermined number of optodes, i.e., light sources/emitters and detectors, are placed on the subject’s head, covering a specific region or the whole brain, called montage. The choice of the montage is determined by the nature of the experiment and based on the prior knowledge of the activation region in the brain for a given stimulation [12]. For, e.g., experiments involving motor tasks, the pairs are placed and the hemodynamic response is acquired from the motor cortex (Figure 8), whereas for higher cognitive functions or mental imagery, the emitter/detector pairs are placed around the prefrontal cortex (Figure 9).

**Figure 10:**
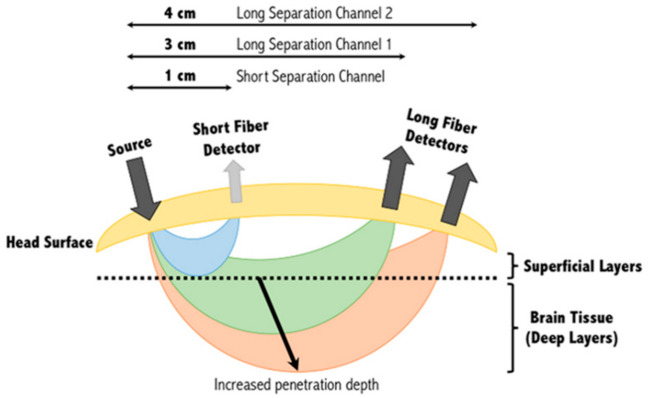
The image shows that as the distance between the emitter–detector pair grows, the deeper the signal penetrates. However, as you increase the depth imaged, the weaker the signal becomes; therefore, the optimal pair distance is around 3 cm. (Reprinted from [13]).

The optimal separation between the emitter–detector pairs is about 3 cm and the penetration depth is half the separation of the source–detector distance (Figure 10). As the distance between emitter–detector pairs increases, the penetration depth also increases, but results in a weaker signal. A separation below 1 cm may only penetrate up to the skin layer, and a separation of over 5 cm results in weak signals. Typically, the emitter–detector pairs are positioned 3 cm apart. Hence, keeping the distance uniform for all the probes to the target region is crucial to correlate the results. The number of channels used for a given montage and the sampling frequency at which the data are acquired are vendor-specific. The sources emit near-infrared light in the optical window of 700–900 nm, which is absorbed by the chromophores, oxyHb and deoxyHb, whereas it is transparent to the skin tissues and bones.

The relative changes in the hemoglobin (Hb) concentration are thus calculated by the differences in the absorption spectrum of oxyHb and deoxyHb. The quantification of oxyHb and deoxyHb depends on the type of the source used for light illumination. In general, there are three techniques used for light illumination, viz., (1) a constant light illumination through the continuous wave (CW) technique, which gives the measure of the light attenuation, (2) the frequency domain technique in which the source delivers the intensity-modulated light, which gives the measure of the light attenuation and the phase delay, and (3) the time domain method in which the light is illuminated as short pulses and the detector detects the shape of the scattering pulse. Among the above methods, fNIRS systems based only on the time and frequency domain techniques can be used to measure the absolute concentration changes in oxyHb and deoxyHb. The collected fNIRS signals consist of the raw optical density (OD) data. By using the modified Beer–Lambert law, the optical density signals are converted into changes in the oxyHb and deoxyHb concentrations [14].

### 2.2. Feature Extraction

**Figure 11:**
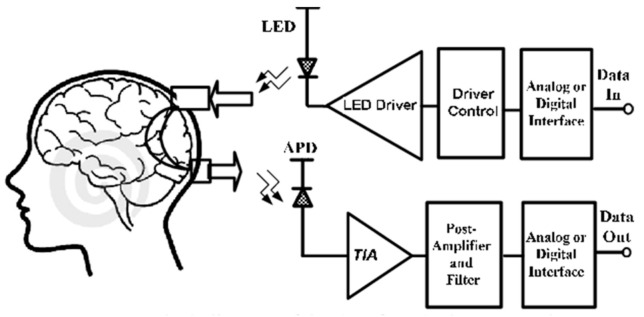
A diagram of fNIRS emitter–detector pair transceiver. (Reprinted with permission from ref. [20], Copyright 2011 IEEE).

Pre-processing and feature extraction are followed by signal acquisition, in which the acquired fNIRS data are cleaned by the removal of various artifacts [15,16,17]. The sources associated with the noise in the data are instrumental, physiological, and the noise associated with experimental error. Instrumental noise is signals that appear in the environment or the hardware itself [18]. Instrumental noise typically comes in the form of a constant high-frequency signal. Thus, passing the data through a low-pass filter will remove the high-frequency signals. Another method of removing noise is by attaching short separation channels to the fNIRS cap. These channels read data from the scalp, thus allowing the elimination of this kind of noise within the data [19]. Experimental errors, such as an accidental shift in the placement of nodes, are difficult to mitigate; however, some methods are proposed, viz., the Wiener filtering-based method, eigenvector-based spatial filtering, wavelet-analysis-based methods, and Savitzky–Golay-type filters. Finally, there is also physiological noise such as the patient’s heartbeat. Using band-pass filtering (which removes known physiological noise that does not overlap with the hemodynamic response), ICA, and PCA, these physiological noises can be removed. The complete setup involves the placement of various components so as to achieve maximum accuracy with the least amount of noise (Figure 11).

Generally, feature extraction utilizes heuristics and uses common features to see the similarities [21,22,23]. The most common features utilized to distinguish the hemodynamic response are signal mean, signal slope, signal variance, amplitude, skewness, kurtosis, and zero crossing.

For the BCI to generate the right output, the features of the hemodynamic response must be interpreted correctly.

Once the raw data are acquired, relevant features are filtered and extracted, making use of time domain, frequency domain and time–frequency domain methods. The most prominent of these is the use of the time–frequency domain for feature extraction purposes, which includes Wavelet Transform and Hilbert Transform. We discuss these two feature extraction processes in this paper.

#### 2.2.1. Wavelet Extraction

**Figure 12:**
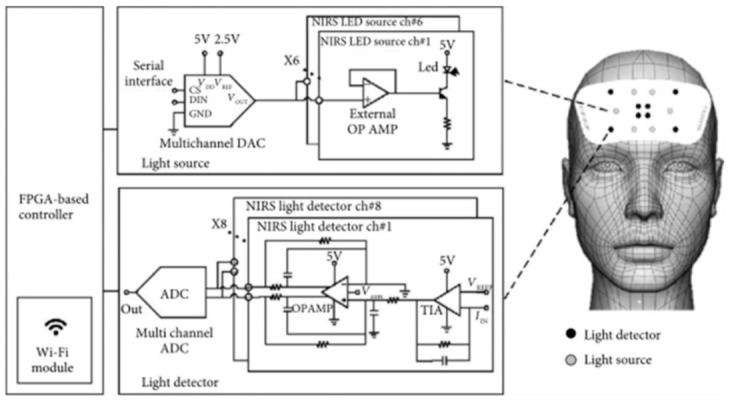
A proposed system for fNIRS utilizing deep forest algorithm. (Reprinted from [32]).

Wavelet-based pre-processing techniques are employed for the estimation of relevant features where multiple aspects of signal quality are taken into account. Scaling functions are used for reconstruction, whereas the order of the wavelets is used for decomposition purposes [24]. Primarily, the signal is decomposed using wavelet functions and then thresholding operations are conducted in scale to eliminate noisy artifacts. Using the resultant wavelet coefficients, subsequent classification is performed to maximize the accuracy and specificity. The classifiers used the most are back propagation neural network (BPNN), linear discriminant analysis (LDA), and support vector machine (SVM). Out of these, the most optimal classifier is chosen after a pre-determined set of trials for further analysis [5]. One study reported that neuronal activity is directly proportional to oxyHb levels, where the circulatory system plays an important part in rapid signal firings [25]. This blood oxygen level dependence (BOLD) signal acts as a base standard estimate of the change in blood flow and is utilized in both fNIRS as well as fMRI measurements [26]. This measure provides a common denominator so that both fMRI and fNIRS can be used in tandem for brain scans so as to obtain the benefits of both techniques [27]. Moreover, BCI techniques based on fNIRS and fMRI suffer from a delayed response from the output system as the hemodynamic response peaks around 6 seconds from the stimulus onset [28,29]. However, the delayed response is addressed by using a multivariate pattern classification technique, which reduces the latency by 50% and facilitates the fNIRS-BCI setup to be implemented in a real-time neurofeedback application [30]. This is achieved by implementing multi-channel DAC inputs and ADC outputs (Figure 12) [31].

#### 2.2.2. Hilbert Transform

Hilbert transform performs orthogonal phase-shifting operations on a function, notwithstanding its frequency. This is most useful in modulating signals with single-sided Fourier transforms. However, it has high latency for low-frequency modulations given its long delay and implies a computational overhead for maintaining historical data of the signal [33]. Nevertheless, it is frequently used for the modulation of bandpass signals in BCI setups [34,35,36].

Once the mandatory steps of data extraction and feature selection are completed, filtered clean data and unwanted artifacts are obtained that can be used for further analytics. Post feature extraction involving normalization and noise reduction, the clean data are passed through various classification approaches. These classifiers can be linear, Bayesian, nearest neighbors, discriminant analysis (LDA), or Artificial Neural Networks (ANNs). The linear model is the simplest, with a weighted approach having signal parameters attached to each other in a linear fashion, whereas the Bayesian model is a probabilistic model where variables are proportionated via a directed acyclic graph. LDA is applied via an n-k support hyperplane with a penalty for miscalculations. ANNs have a multi-layered structure and employ likelihood algorithms with multivariate generative distribution to their weights. Here, we discuss the various ANNs in detail.

### 2.3. ANN Classification Approaches

Given the holistic nature of the decision-making process of an ANN that is based on the predictability of input features, a multilayer feed forward perceptron can be trained as a non-linear classifier using the generalized backpropagation (BP) algorithm, in which momentum is chosen as a standard training principle to speed up convergence while maintaining generalization [36,37]. Bipolar sigmoid functions are used as decision functions for hidden layers, whereas unipolar sigmoid functions are used for output layers. These processes help achieve the most optimal NN classifier that, in turn, is trained and tested using the feature sets under selection.

A series of discrimination algorithms may be utilized to aid in computing and translating the data [5]. Some of these include linear discriminant analysis (LDA), support vector machine (SVM), artificial neural network (ANN), and hidden Markov model (HMM) [38]. LDA is an algorithm that, due to its simplicity and low computational requirements, is utilized most often. LDA works by separating two classes into separate categories and identifying them as different outputs. It is a form of supervised learning where the results are known, and the algorithm learns from the data. Support vector machine (SVM) is also a form of supervised learning, but it tries to maximize the differences between training material. Thus, it enhances generalization by reducing errors on training materials.

#### 2.3.1. Probabilistic Neural Networks

This approach has been in use within the framework for pattern recognitions in various waveforms. It has been derived from the radial basis function (RBF) network, which, in turn, is a bell-shaped function following the parameter in a non-linear manner. The most important advantage of PNNs is their speed, which happens to be many times faster than BP networks and is able to match Bayes optimal results fairly easily with much better performance. The relative speed is achieved on account of the core framework of matrix multiplications that makes the process inherently fast [39]. In this approach, weights are never “trained” but are instead assigned directly and are not altered afterwards. This enables the performance of analytics in real time.

#### 2.3.2. Support Vector Machines

Support Vector Machine (SVM) is a classification algorithm that utilizes the principle of structural risk minimization that utilizes a high-dimensional feature space wherein waves are charted using non-linear mapping fundamentals and subsequent linear regressions are conducted on each planar space. To obtain a sense of linearity, a hyperplane is traced using maximizing projection between the means and classes that bisects the latter and optimizes the margin of separation. This provides generalization ability to the learning algorithm [40].

Both LDA and SVM are linear classifiers. Artificial neural networks (ANNs) are systems that mimic human and animal brains in discriminating points of data by taking environmental factors into account. They work via many artificial neuron pathways and look for groupings to classify the data. The Hidden Markov Model utilizes the probability of seeing distinctions to classify the data. Both ANN and HMM are non-linear classifiers. Such discrimination systems are employed in data extraction for convenient utilization.

The most important goal that SVM regression chases is to minimize the error parameterized by the hyperplane that maximizes the margin. However, the idea is to make sure that the error is always kept within tolerance levels. This means that SVM excels in both Gaussian and radial datasets and performs quite well for non-linear data without requirement of any form of guesswork about their functional form. Since data segregation is performed with the maximum possible margin, the resulting model has better stability and can deal with inconsistencies such as noise or training bias [41].

## 3. Challenges in fNIRS-BCI

**Figure 13:**
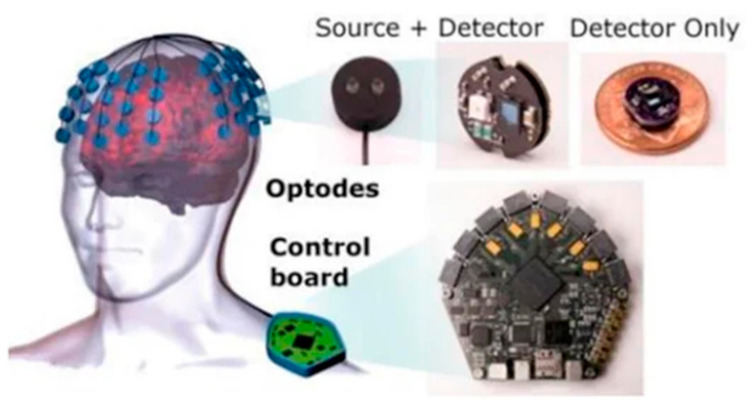
An example of the equipment needed for a fNIRS-BCI system. The hardware can be modular and non-invasive, important for the daily use of the user. (Reprinted from [44]).

There are many advantages in the use of BCI based on fNIRS. One of the major advantages is that it is portable, while imaging techniques such as MRI and MEG require a large room with protective shields to minimize external interference. In comparison to EEG-BCI, fNIRS does not require any application of gel on the subject’s scalp [42]. An fNIRS-based BCI is sizeable and can easily be implemented in experiments simulating real-life scenarios [43]. Since fNIRS measures the changes in the endogenous Hb, no external agent is required for signal acquisition. Finally, fNIRS machines are non-invasive and avoid the need for surgery or the implantation of devices for their implementation (Figure 13).

**Figure 14:**
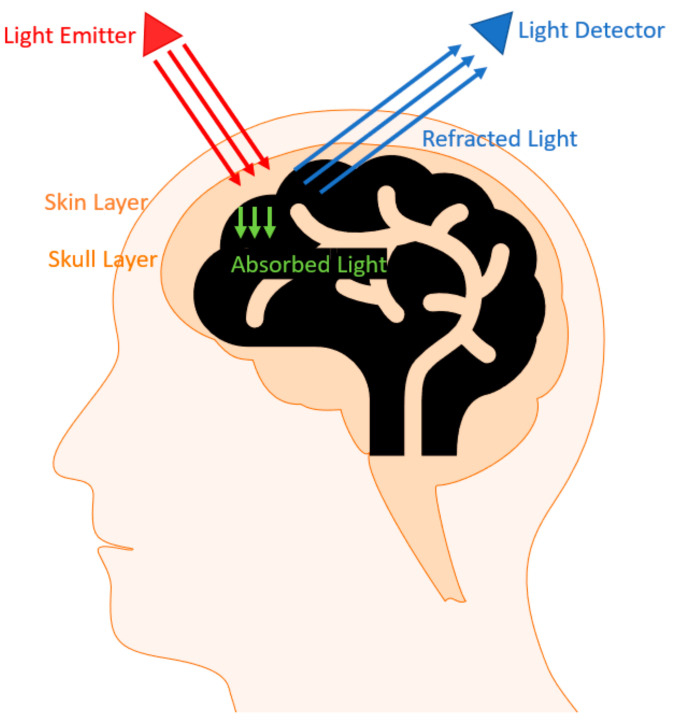
Schematic diagram showing the non-uniform spacing between skull and brain owing to different composition of tissues.

Apart from these general technical challenges, researchers have to also deal with session and subject-wise differences in classifying the working memory-based varying workloads. These problems are classified as domain adaptation in the machine learning step where data from separate subjects as well as sessions are classified as belonging to different domains [30,32,33,34,35]. The subsequent changes in the distribution of wavelets across separate domains, notwithstanding whether emanating from the subject or captured in different sessions, are classified as domain shift [36]. This debilitation limits the knowledge learned that can be shared across domains as well as sessions. This issue is partially addressed by the latest developments in optimal transport methods and metric measure space alignment [37,38]. Figure 14 shows a schematic diagram showing the non-uniform nature of the brain–skull spacing.

**Figure 15:**
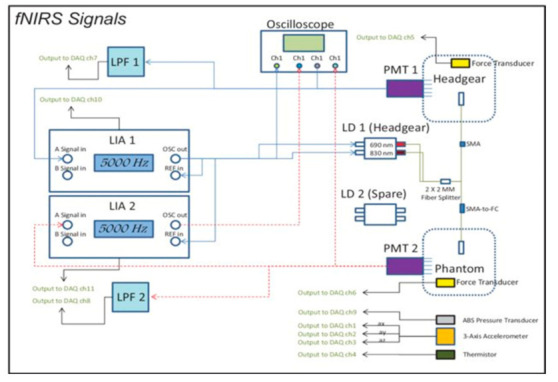
Schematic of fNIRS experimentation under different levels of gravity for the accuracy of signal generation. (Reprinted from [45]).

While there are many advantages, there are also disadvantages to fNIRS-BCI. fNIRS is based on a relatively slow hemodynamic response, and due to the limited number of sensors used, the spatial resolution is low. The non-uniform nature of the skull remains a challenge in positioning the sensors, which introduces some error in the measurement [39]. Multiple force transducers are used to amplify the signal and multichannel filters and multiplexers are set up in parallel to maximize output (Figure 15).

These challenges can be overcome with a combined imaging system. An EEG-BCI uses the post-synaptic potential that occurs in the brain to monitor and detect areas in the activated region [42]. In terms of hardware, EEG utilizes a cap of electrodes as well as a gel, which is applied for optimal contact between the electrode and scalp. While EEG data are of low spatial resolution, it is a non-invasive technique, which makes it feasible in BCI applications. An MEG-BCI relies on the tiny magnetic field, in the order of femto Tesla, generated in the brain as a result of the post-synaptic potential due to the given stimuli [39]. However, MEG systems are placed in a magnetically shielded room to reduce the environmental noise, thereby making it difficult in BCI-based applications. On the other hand, MEG offers a higher spatial resolution and a non-invasive mode of data acquisition. Finally, the fMRI-BCI uses changes associated with blood flow to detect brain activity for signal acquisition. MRI uses a large magnet placed in a shielded room. Similar to MEG, it delivers high spatial resolution data and is non-invasive, but not portable. These are some imaging systems that can be used in conjunction with fNIRS-BCI. However, it must be noted that most of these techniques are dependent on the employment of auxiliary reference signals such as accelerometery or extra-optical channels. This leads to the insertion of implicit assumptions in the characteristics of motion artifacts and subsequently filtered fNIRS signals. Multiple approaches guided by statistical signal processing methods including adaptive filtering, independent component analysis (ICA), and time–frequency analysis have been employed to formulate algorithms that can help fix for motion artifacts in fNIRS signals [12,41,46,47,48,49,50,51,52,53,54].

## 4. Machine Learning in fNIRS

Machine learning is a set of computation algorithms that allows for better classifying and sorting the data. With machine learning, it is possible to streamline and refine the feature extraction process as well as combine different modalities together to obtain better precision. A machine learning algorithm learns from prior data and then the experimental data are fed in for the analysis and classification. There are two types of classifiers: linear and non-linear. Linear classifiers mark the data in pre-set categories, while non-linear classifiers work better when there is not a clear indication of separate categories or features to extract.

During the feature estimation step, a wavelet-based pre-processing technique is employed, where there are different parameters of signal quality for the estimation of task-relevant signals and the elimination of artifact interference that causes undue noise. Hence, for the detection of useful signals, the input is passed through filters for noise reduction, decomposed using candidate wavelet functions, and then soft thresholding is performed using MiniMax, hybrid, or SURE methods [5,54]. Wavelet functions are classified as non-orthogonal, orthogonal, and biorthogonal families of wavelets. The first one of these is also known as continuous wavelets since they form the continuous counterpart of orthogonal wavelets, with notable examples being Morlet and Mexican hat wavelets where is latter is a normalized second derivative of a Gaussian smooth function. The latter class of wavelets enable the non-redundant part of the input waveforms and are defined by the scaling and wavelet functions. Prominent examples composing this class of waveforms are Meyer and Shannon wavelets. These waveforms are symmetric in nature and lack any form of compact support. The last class of wavelets are in fact an interpretation of the orthogonal waveform acting as a generalization of the approximation signals. These waveforms are asymmetric in nature and never induce phase shifts in coefficients. Important examples of this waveform are B-spline wavelets, which are semi-orthogonal with support for compact wavelets [5].

## 5. fNIRS-BCI Applications

fNIRS-BCI systems have a multitude of applications, especially in the clinical domain. BCI finds its application in areas such as communication, motor function, neuroergonomics, neurorehabilitation, environmental control, and entertainment [55]. In the purposes of communication, BCI can aid those who suffer from Amyotrophic Lateral Sclerosis (ALS), spinal cord injury, or locked-in syndrome (LIS). An example of BCIs being used in the aid of communication is in binary communication. Naito et al. (2007) and Naseer et al. (2014) created a system of having subjects answer yes or no by performing mental arithmetic [10,56]. A relaxed mind would result in a “no,” and a mind performing mental arithmetic would result in a “yes.” Another idea for aid in more complex communication is to move a cursor left and right to select a letter, as proposed by Sitaram et al. (2007) [57]. Other than enabling a subject to communicate with the outside world, the creation of BCI setups to enable a subject to restore mobility is another interesting area where current research is undertaken. The time-resolved fNIRS-BCI has been demonstrated in the application of mental communication in healthy volunteers, with the possibility of potential clinical applications in patients with brain injury. fNIRS-BCI has also been employed in the effect of yoga meditation practice on young adults’ inhibitory control studies. Another application lies in increasing motor cortex activation during grasping via novel robotic mirror hand therapy.

In the application related to motor functions, disabled patients can benefit from BCI by having prosthetics controlled by the brain. Currently, there is development on enhancing the accuracy and speed of these BCI-controlled prosthetics and wheelchairs by improving the speed of data acquisition, processing, and transporting to execute the function. There have been many improvements in the area of wearables as well.

Further, the application of fNIRS-BCI in neuroergonomics promises that mental workload and conditions can be performed in real time. Three different levels of workload have been identified in air traffic control tasks [58], attention deficit tasks [59], and the detection of drowsiness in drivers [60] has been demonstrated using fNIRS-BCI. For the restoration of cognitive and motor functions in stroke patients by regulating brain activity, a neurofeedback process has been shown in [61], in which the hemodynamic response is controlled by the subjects. Further fNIRS-BCI systems may assist motor-disabled patients in controlling electrical appliances. BCIs can help those who suffered through a loss of motor or cognitive function due to a stroke or spinal cord injury through feedback to help regain the self-regulation of brain activity.

## 6. Software for fNIRS-BCI

In this section, we will discuss some of the software that supports fNIRS-BCI applications.

Homer2 is the most popular software and the first to be developed for the visualization of measurements and image construction of fNIRS data, and it currently supports the calculation of the optical forward model (sensitivity matrix) from a homogeneous, semi-infinite slab geometry [22]. The functional responses can be obtained by block averaging on a deconvolution model based on the estimation of an FIR impulse response. This process makes use of an ordinary least squares fit of the data.

OpenVibe is an open-source software that allows for connections to virtual reality (VR) [33]. It is modular and reusable, allowing a wide range of people to utilize it and reduce how long something takes to be developed. It is portable as it can be installed in any Windows or Linux machine and is independent of any hardware. Another accomplishment this software boasts is its connection to VR. This software can be integrated with many VR systems to allow for neurofeedback.

Another software is BCILAB. BCILAB is more focused on experimental BCIs and neuroscience [15]. It utilizes MATLAB as its base platform. It has a large toolbox for methods of BCI approaches with emphasis on evaluation tools and flexibility. There is extensive documentation allowing for an easier learning curve. It is one of the most popular BCI software programs currently in use for fNIRS.

NICA is a novel toolbox for fNIRS calculations and analyses based on MATLAB that enables the processing and visualization of fNIRS data, including different signal processing methods for physiological artifact correction coming from noise generated by systemic influences and physiological artifacts [62]. This toolbox has been developed for reading fNIRS data and currently supports NIRScout 1624 measurements and its complementary recording software NIRStar.

## 7. Future Directions

**Figure 16:**
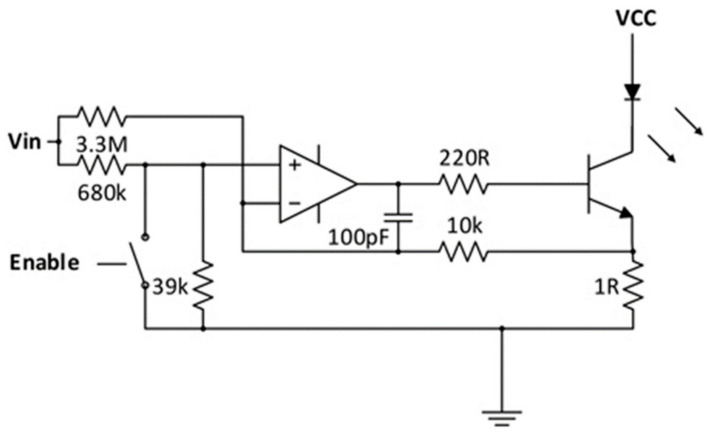
Current regulator circuit. (Reprinted from [64]).

**Figure 17:**
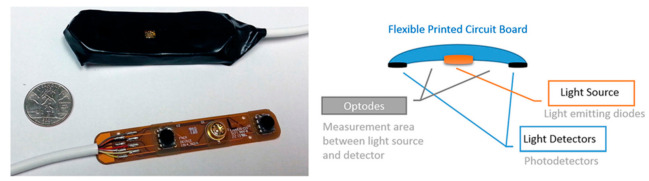
A small scalable example of fNIRS sensor pad. The size shows the convenience of fNIRS to not interfere with everyday life. This model contains two optodes. (Reprinted from [64]).

The current modalities are not completely portable and functional for purposes of BCI technology such as neuro-prosthetics [63]. In contrast, fNIRS has been proven to be easily adaptable into portable systems given its simple circuit design (Figure 16). Future developments of BCI are currently being focused on the development of three main domains, viz., signal acquisition, hardware, and reliability. Improvements in these three areas could improve the quality of data for a better reading and improving the prediction rates for BCI technology. With improvements to the ability to gain data, we may see more functional uses in everyday life. Additionally, the hardware must be durable to ensure usability in a multitude of environments as well as be able to last for decades. In order to ensure that the hardware is long-lasting, one must also look at the power system. Finally, it must be ensured that these devices are safe for human use. There has already been a good head start in this direction where portable setups have been created (Figure 17).

However, the technology must also be improved in the means of reliability. Currently, the machines are not reliable enough to be used for real applications. Current research is looking at dual fNIRS and EEG in data acquisition for BCI [65]. It has been reported that these hybrid systems are more accurate than EEG alone or fNIRS alone. With a rise in reliability, the use of these machines can be improved to help a variety of causes.

This strategy is not just limited to imaging Hb; it can also be extended for imaging other targets of the brain by designing fluorescent probes that are pre-delivered before adopting NIR imaging, which can be combined with the existing functional imaging as a multiplex approach to collect more information regarding the health status of the brain, especially in the case of Alzheimer’s, Parkinson’s, and other brain diseases.

## 8. Other Neuroimaging Modalities

There are many neuroimaging tools such as EEG, MEG, ECoG (Electrocorticography), and fMRI. While EEG and MEG offer higher temporal resolution, ECoG is invasive. A multimodal approach in which combinations of these techniques with fNIRS may allow for a higher accuracy in data acquisition thereby permits a higher rate of success in feature extraction while keeping them portable. However, one of the advantages of fNIRS is that it can be complementarily combined with other modalities where its instruments do not create any fresh artifact noise in the readings relevant to other modalities in question and vice versa [66]. The most common combination pursued is fMRI–fNIRS, which takes benefit of the fact that the fMRI setup is not able to conduct experiments with the subject in a sitting or standing position [67]. Moreover, it does not allow access to the working body parts for the researcher to take control readings in real time. Nevertheless, fMRI is able to provide extremely accurate readings. To compensate for these problems while still not forgoing the benefits, an fNIRS setup can be coupled together [68] with the existing setup that can conduct real-time brain training in a functional position with an acceptable spatial resolution of about 1 cm and a penetration depth of 1.5–2.5 cm, as stated earlier [69]. fNIRS has also been combined with the EEG modality, which is able to provide a distinct neuromonitoring platform to explore neurovascular coupling mechanisms [70]. A modified form of fNIRS called broadband-NIRS has been specifically used for this purpose with the use of Finite Impulse Responses functions within the General Linear model. It has been demonstrated that such an implementation is able to measure hemodynamic and metabolic activity in the occipital cortex [70].

## 9. Conclusions

In this paper, we have reviewed the use of functional near-infrared spectroscopy in brain–computer interfaces. We discussed that fNIRS is the process of observing the hemodynamic response via near-infrared light-based imaging. We discussed how BCIs work; in summary, after receiving data from the fNIRS, BCIs are programmed to filter out extraneous noise before the data can be extracted for useful features via filtering, feature selection, and post-processing. Through such feature extraction methodologies, an appropriate response is executed. Additionally, we discussed the development of hardware over time, chronologically from the discovery of NIRS, to observe hemoglobin levels up to the development of small, wireless fNIRS devices. The application of BCI was briefly discussed, and we learned about the various uses for such devices. We learned that the devices can be used for communication by having the patient answer binary questions with the thought of mental arithmetic. They can also be used to help amputees regain motor functions through prosthetics that are controlled by BCI systems. Finally, fNIRS-BCI can be used in neurorehabilitation by giving the patient feedback. We also looked at the advantages and disadvantages of fNIRS-BCI systems. The system was found to be useful in that it is extremely portable. The system was also relatively cheaper than most other imaging systems. fNIRS is also non-invasive and thus can be used easily. The disadvantages we found were speed limitation due to delay in the hemodynamic response and the fact that the system is not completely accurate and not ready yet for real applications. Finally, we looked at future developments that are being worked on. Future developments include making the system more user-friendly. For non-scientific or medical use of this system, there needs to be an improvement in making it easier to set up and use. There is also research looking to make the system more reliable. Currently, there are developments on making a hybrid fNIRS–EEG system for BCI.

## Figures and Tables

**Table 1:** A timeline overview of the development of fNIRS and fields of application. (Reprinted from [1]).

Year	Major Events
1977	Jöbsis demonstrates the possibility to detect changes in adult cortical oxygenation during hyperventilation by near-infrared spectroscopy.
1985	First NIRS clinical studies on newborns and adult cerebrovascular patients (Brazy; Ferrari).
1989	First commercial single-channel CW clinical instrument: NIRO-1000 by Hamamatsu Photonics, Japan.
1991/1992	First fNIRS studies carried out independently by Chance, Kato, Hoshi, and Villringer by using single-channel instruments.
1993	Publication of the first 6 fNIRS studies.
	Simultaneous monitoring of different cortical areas by 5 single-channel instruments (Hoshi).
1994	First application of fNIRS on subjects affected by psychiatric disorders by using a single-channel system (Okada).
	Hitachi company (Japan) introduces a 10-channel CW system (Maki).
	First simultaneous recording of positron emission tomography and fNIRS data (Hoshi).
1995	First evidence of a fast optical signal related to neuronal activity (Gratton).
	First two-dimensional image of adult occipital cortex activation by a frequency domain spectrometer (Gratton).
1996	First simultaneous recording of fMRI and CW fNIRS data (Kleinschmidt).
	First simultaneous recording of fMRI and TRS fNIRS data (Obrig).
1998	First application of fNIRS on newborns using a commercial single-channel CW system (Meek).
	First images of the premature infant cortex upon motor stimulation by using a CW–fNIRS prototype (Chance).
	First application of the Hitachi 10-channel system in clinics (Watanabe).
1999	First introduction of a 64-channel TRS system for adult optical tomography (Eda).
	First introduction of a 32-channel TRS system for infant optical tomography (Hebden).
	First optical tomography TRS images of the neonatal head (Benaron).
	Introduction of the first compact 8-channel TRS system (Cubeddu).
	TechEn company (USA) starts to release its first fNIRS commercial system.
2000	Hitachi company starts to release its first commercial system: (ETG-100, 24 channels).
2001	First fNIRS study using a single-channel CW portable instrument and telemetry (Hoshi).
	Shimadzu company (Japan) starts to release its first commercial system: (OMM-2001, 42 channels).
	ISS Inc. (USA) starts to release the frequency domain system: Imagent (up to 128 channels).
	First three-dimensional CW tomographic imaging of the brain (DYNOT, NIRx Medical Technologies, US) (Bluestone).
2002	Hitachi company starts to release the ETG-7000 (68 channels).
2003	Hitachi company starts to release the ETG-4000 (52 channels).
	Artinis company (The Netherlands) starts to release the Oxymon MkIII (up to 96 channels).
2004	Shimadzu company (Japan) starts to release the NIRStation (64 channels).
	First simultaneous recording of DC-magnetoencephalography and CW fNIRS data (Mackert).
2005	Hitachi company starts to release the ETG-7100 (72 channels).
2007	Shimadzu company starts to release the FOIRE-3000 (52 channels).
2009	fNIR Devices company (USA) starts to release a wearable 16-channel system for adult PFC measurements.
	Hitachi company starts to release a battery-operated wearable/wireless 22-channel system for adult prefrontal cortex measurements.
2011	NIRx Medical Technologies company (USA) starts to release a battery-operated wearable/wireless 256-channel system for adult frontal cortex measurements.

## Data Availability

Not applicable.
